# LncRNA HULC mediates radioresistance via autophagy in prostate cancer cells

**DOI:** 10.1590/1414-431X20187080

**Published:** 2018-04-23

**Authors:** Changxuan Chen, Kaizhen Wang, Qian Wang, Xin Wang

**Affiliations:** 1 Department of Urology, Tengzhou Central People's Hospital, Affiliated to Jining Medical College, Tengzhou, China; 2 Department of Traumatology, Tengzhou Central People's Hospital, Affiliated to Jining Medical College, Tengzhou, China

**Keywords:** Prostate cancer, lncRNA HULC, Irradiation, Autophagy, mTOR

## Abstract

Prostate cancer (PCa) is the second leading cause of cancer death in men. Irradiation is one of the available options for treatment of PCa, however, approximately 10–45% of PCa are resistant to irradiation. We aimed to explore the role of long non-coding RNA highly upregulated in liver cancer (HULC) in the sensitivity of PCa cells to irradiation. Survival rate, cell apoptosis, cycle, expressions of related proteins, and caspase-3 activity were assessed to explore the effects of HULC on sensitivity of PCa cells to irradiation. Expression of HULC in DU-145, PC3, LNCaP, and RWPE-1 cells was determined and the influence of HULC on DU-145 cells was explored. Then, PC3 cells aberrantly expressing HULC were implanted into NOD-SCID mice for tumor xenograft study. Changes of autophagy after aberrant expression of HULC *in vivo* and *in vitro* were tested. Furthermore, the interacted protein of HULC and involved signaling pathway were investigated. In PC3 and LNCaP cells under irradiation, survival rate and cell cycle were decreased and apoptosis was increased by HULC knockdown. HULC knockdown arrested PC3 cells at G0/G1 phase. DU-145 was sensitive to irradiation, and resistance to irradiation of DU-145 cells was enhanced by HULC overexpression. Moreover, HULC knockdown enhanced the sensitivity of PC3 xenografts to irradiation. HULC knockdown promoted autophagy through interaction with Beclin-1 and inhibition of mTOR, resulting in increased apoptosis. HULC knockdown improved sensitivity of PCa cells to irradiation both *in vivo* and *in vitro*. HULC suppressed Beclin-1 phosphorylation, thereby reduced autophagy, involving the mTOR pathway.

## Introduction

Prostate cancer (PCa), the most common cancer in men, account for approximately 26% of newly diagnosed cancer cases in 2015 in the United States (1). In men, PCa is the second leading cause of deaths related to cancer, which leads to 1–2% of deaths (2). Surgery, radiation, and hormone therapy are widely accepted to be pillars for cancer treatment (3). By utilizing prostate specific antigen (PSA) screening, an overwhelming majority of PCa can be diagnosed at an early stage, making radiotherapy a standard treatment modality for PCa (4–6). Resistance to radiation, present in approximately 10–45% of PCa, is a crucial contributing factor, which largely influences the outcome of radiotherapy (6,7). Considering that enhanced doses of radiation may induce side-effects on normal cells around the tumor, increasing the sensitivity of PCa cells to radiation becomes a potential therapy for PCa.

Long non-coding RNAs (lncRNAs) are transcripts with more than 200 nucleotides and have been recently identified to participate in the development and progression of PCa (8). Highly upregulated in liver cancer (HULC) lncRNA, which is transcribed from chromosome 6p24.3, was initially recognized as a key molecule in human liver cancer (9). Recently, HULC has been identified to be a novel biomarker in diverse cancers, and it acts as an oncogenic factor (10,11). A previous study has proposed that HULC silence could inhibit angiogenesis in human gliomas (12). Another study proved that HULC promoted progression of colorectal carcinoma (13). However, the role of HULC in PCa remains unclear and the related literature is limited.

Autophagy, a process of cellular self-eating, is a catabolic process that delivers cellular proteins and organelles to lysosomes, followed by degradation (14). Autophagy plays a key role in metabolism control in both normal and diseased cells. Even though the process of autophagy may improve cell survival under harsh conditions, in many cases, autophagy can induce autophagic programmed cell death, which is also termed as type II cell death (15,16). Autophagic and apoptotic pathways are interconnected, and the loss of homeostatic balance between these two pathways may lead to cellular death (17). Accumulating evidence has shown that autophagy can facilitate cell death (18). Moreover, the effects of HULC on autophagy are controversial in different cancer cells. In epithelial ovarian carcinoma cells, it has been reported that HULC overexpression reduces autophagy through the down-regulation of Beclin-1 and microtubule-associated protein 1 light chain 3B (LC3B)-II (19). Conversely, another study has reported that HULC can trigger autophagy in hepatocellular carcinoma cells (20). The specific influence of HULC on autophagy in PCa cells remains unclear.

The goal of this study was to explore the functional role of HULC in PCa. Specifically, the expression of HULC in PCa cells and the effects of its aberrant expression on PCa cells under irradiation *in vivo* and *in vitro* were investigated. Considering that altered autophagy of cancer cells may affect radiation resistance, the alterations of autophagy after aberrant expression of HULC as well as underlying mechanisms were also explored.

## Material and Methods

### Cell culture and X-ray irradiation

Three PCa cell lines, including PC3, LNCaP, and DU145 cells as well as normal human prostate epithelial cells (RWPE-1) were obtained from American Type Culture Collection (USA). PCa cells were maintained in RPMI 1640 medium (Gibco, USA) containing 10% fetal bovine serum (FBS; Gibco) and 1% penicillin/streptomycin (Invitrogen, USA). RWPE-1 cells were cultured in Keratinocyte Serum Free Medium (K-SFM; Gibco) supplemented with 1% penicillin/streptomycin. Cells were maintained in a humidified incubator with 5% CO_2_ at 37°C. For mammalian target of rapamycin (mTOR) inhibition, cells were incubated with Torin 1 (250 nM; Selleck, USA).

The Shimadzu X-TITAN 225S X-ray generator (Shimadzu, Japan) was employed to deliver a dose of radiation (6 Gy), with a dose rate of 2 Gy/min. Monolayer cells with logarithmic growth were exposed to X-ray at ambient temperature, and the cells in control groups received sham treatment without irradiation. After irradiation, the cells were collected immediately for subsequent experiments.

### Stable cell transfection and RNA interference

Full-length HULC sequences were ligated into pEX-2 plasmid (GenePharma, China) and the resultant plasmid was referred to as pEX-HULC. For HULC knockdown, short-hairpin RNA targeting human HULC was sub-cloned into pGPU6/GFP/Neo plasmid (GenePharma) and the resultant plasmid was referred to as sh-HULC. The pGPU6/GFP/Neo plasmid carrying a non-targeting sequence was referred to as sh-NC, acting as the negative control of sh-HULC. Cell transfection was performed using Lipofectamine 2000 reagent (Invitrogen) according to the manufacturer's instructions. Stably transfected cells were generated by transfection of pEX-HULC, pEX-2, sh-HULC or sh-NC, followed by sequential selection with 0.5 mg/mL G418 (Sigma-Aldrich, USA).

### Apoptosis assay

Cell apoptosis was assessed by dual staining with fluorescein isothiocyanate (FITC)-conjugated Annexin V and propidium iodide (PI). Briefly, after treatments, cells were washed in phosphate buffered saline (PBS) and were resuspended in binding buffer. Then, cells were treated with Annexin V-FITC and PI according to the instructions of the Annexin V-FITC/PI apoptosis detection kit (Beijing Biosea Biotechnology, China). The percentage of apoptotic cells was tested using a FACScan flow cytometer (Beckman Coulter, USA) and analyzed using FlowJo software (Tree Star, USA).

### Quantitative reverse transcription PCR (qRT-PCR)

Total RNA was isolated from cells by using TRIzol reagent (Invitrogen) according to the supplier's instructions. Reverse transcription from RNA to cDNA and quantitative PCR were performed using One Step SYBR¯ PrimeScript™ RT-PCR Kit (Perfect Real Time; Takara, China) following the manufacturer's protocol. The conditions were programmed as follows: 5 min at 42°C, 10 s at 95°C, followed by 40 cycles at 95°C for 5 s, and 60°C 30 s. Primers for qRT-PCR were: HULC sense, 5′-ACTCT GAAGT AAAGG CCGGA-3′, HULC antisense, 5′-TGCCA GGAAA CTTCT TGCTT G-3′; GAPDH sense, 5′-CAGCC AGGAG AAATC AAACA G-3′, GAPDH antisense, 5′-GACTG AGTAC CTGAA CCGGC-3′ (Sangon, China). Relative expression of HULC was calculated according to the 2^-ΔΔCt^ method (21), normalizing to GAPDH.

### Western blot analysis

Proteins of cells and tissues were extracted in RIPA lysis buffer (Beyotime Biotechnology, China) supplemented with a cocktail of protease inhibitors (Roche, USA). The amount of proteins was determined by BCA™ Protein Assay Kit (Pierce, USA), and then equivalent proteins were loaded and separated by SDS-PAGE gels. Afterwards, proteins were blotted to polyvinylidene difluoride (PVDF) membranes and the membranes were blocked by 0.5% skimmed milk. Membranes were then incubated with diverse primary antibodies against Bax (ab182733), active caspase-3 (ab49822), proliferating cell nuclear antigen (PCNA; ab152112), cyclinD1 (ab134175), LC3B (ab48394), p62/sequestosome 1 (p62; ab207305), Beclin-1 (ab62557), eukaryotic initiation factor 4E binding protein 1 (4E-BP1; ab131453), phosphorylated 4E-BP1 (p-4E-BP1; ab75767), GAPDH (ab181603) (all from Abcam, UK), or phosphorylated Beclin-1 (p-Beclin-1; ABC118, Kenilworth, USA) at 4°C overnight. After rinsing with Tris-buffered saline containing 0.1% Tween 20 (TBST), membranes were incubated with secondary antibody marked by horseradish peroxidase for 1 h at room temperature. Proteins in the membranes were visualized by using a chemiluminescence (ECL) system (Amersham Biosciences, USA).

### Clonogenic survival assay

After treatment, cells were trypsinized, resuspended in pre-warmed complete medium, and seeded into 2 cm^2^ culture dishes (5×10^5^ cells). Then, cells were maintained at 37°C for 1–2 weeks, followed by counting of colonies with more than 50 cells.

### Caspase-3 activity assay

The caspase-3 activity was estimated by using a caspase-3 activity kit (Beyotime Biotechnology) in accordance with supplier's protocol. In brief, cells were lysed, and the cell lysate was mixed with assay buffer and 2 mM caspase substrate, Ac-DEVD-*p*NA, followed by incubation at 37°C for 2 h. Samples were measured with a microplate reader (Bio-Rad, USA) at an absorbance of 405 nm.

### Cell cycle assay

After treatment, cells were washed with PBS and fixed with ethanol on ice. Then, cells were rinsed with PBS again and incubated with buffer containing 50 μg/mL RNase A (Sigma-Aldrich) and 50 μg/mL PI (Beyotime Biotechnology) at 37°C for 30 min. A total of 10^4^ cells were counted and analyzed by using a FACScan flow cytometer.

### Tumor xenograft studies

A total of 56 immunocompromised NOD-SCID mice (male, 6-week-old) were obtained from the Laboratory Animal Center at the Chinese Academy of Sciences (China). To measure the *in vivo* tumor formation of PC3 cells and their response to irradiation, mice were assigned to seven groups (n=8) with or without subcutaneous injection of 10^7^ PC3 cells transfected with sh-HULC, sh-NC, pEX-2 or pEX-HULC, together with irradiation (0 or 6 Gy) at 4 weeks post-injection. The PC3 cells were embedded in Matrigel (BD Biosciences, USA) before injection. Beginning the second week post-injection, tumor growth was monitored weekly, and the tumor length (L) and width (W) were measured. Tumor volume was estimated using the formula (π/6)(LW^2^).

### RNA pull-down

RNA pull-down was carried out with a Pierce™ Magnetic RNA-Protein Pull-Down kit (Pierce) following the manufacturer's protocol. In brief, HULC or control RNA was labeled with biotin after *in vitro* transcription, which was referred to as Biotin-HULC or Biotin-control. Then, labeled RNAs were incubated with Streptavidin Magnetic Beads, and the complex was then incubated with whole cell lysates. Subsequently, the RNA-protein complex was purified via magnetism and analyzed by western blot analysis.

### Statistical analysis

All experiments were repeated three times. The results are reported as means±SD. Statistical analysis was performed using Graphpad Prism 5 software (GraphPad, USA). P-values were calculated using the unpaired two-tailed *t*-test. P<0.05 was considered significant.

## Results

### Sensitivity to irradiation of PC3 and LNCaP cells was reduced by HULC

**Figure 1.**
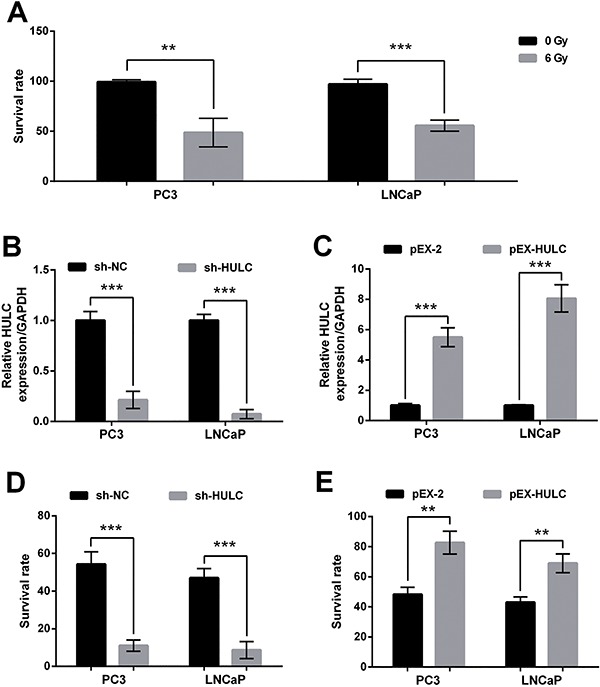
HULC knockdown enhanced the sensitivity of PC3 and LNCaP cells to irradiation. *A*, Survival rate was reduced by irradiation. *B*, HULC was down-regulated by sh-HULC. *C*, HULC was up-regulated by pEX-HULC. *D*, Survival rate after irradiation was further reduced by HULC knockdown. *E*, Survival rate after irradiation was increased by HULC overexpression. Data are reported as means±SD. **P<0.01; ***P<0.001 (*t*-test). HULC: long non-coding RNA highly upregulated in liver cancer; sh-HULC: pGPU6/GFP/Neo plasmid carrying short-hairpin RNA targeting HULC; sh-NC: pGPU6/GFP/Neo plasmid carrying a non-targeting RNA; pEX-HULC: pEX-2 plasmid carrying full-length HULC.

To explore the effects of HULC on sensitivity to irradiation of PCa cells, cells were stably transfected with diverse recombined plasmids. After irradiation, survival rates of PC3 and LNCaP cells were significantly decreased by 6 Gy radiation compared with cells that received 0 Gy radiation (P<0.01 or P<0.001, Figure 1A). Then, qRT-PCR results showed that HULC expressions in PC3 and LNCaP cells were markedly down-regulated by stable transfection with sh-HULC compared with the sh-NC group (both P<0.001, Figure 1B) and significantly up-regulated by stable transfection with pEX-HULC compared with the pEX-2 group (both P<0.001, Figure 1C), indicating HULC was aberrantly expressed after stable transfection. After irradiation (6 Gy), survival rates of PC3 and LNCaP cells with HULC knockdown were significantly lower than the sh-NC groups (both P<0.001, Figure 1D), whereas survival rates in cells with HULC overexpression were remarkably higher than the pEX-2 group (both P<0.01, Figure 1E). These results illustrated that sensitivity to irradiation of PC3 and LNCaP cells was reduced by HULC.

### Effects of irradiation on cell apoptosis and proliferation were enhanced by HULC knockdown in PCa cells

**Figure 2.**
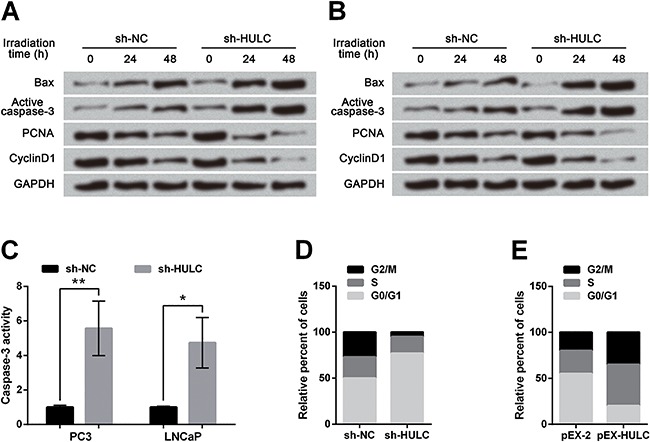
Effects of irradiation on prostate cancer cells were enhanced by HULC knockdown. A dose of 6 Gy irradiation was delivered to cells. Expressions of cell cycle-associated proteins were down-regulated while expressions of apoptosis-associated proteins were up-regulated by HULC knockdown in PC3 cells (*A*) and LNCaP cells (*B*). *C*, Caspase-3 activity was enhanced by HULC knockdown. *D*, HULC silence arrested PC3 cells at G0/G1 phase. *E*, HULC overexpression arrested PC3 cells at S phase. Data are reported as means±SD. *P<0.05; **P<0.01 (*t*-test). HULC: long non-coding RNA highly upregulated in liver cancer; sh-HULC: pGPU6/GFP/Neo plasmid carrying short-hairpin RNA targeting HULC; sh-NC; pGPU6/GFP/Neo plasmid carrying a non-targeting RNA; pEX-HULC: pEX-2 plasmid carrying full-length HULC; PCNA: proliferating cell nuclear antigen.

Results in Figure 2A showed in HULC-silenced PC3 cells that Bax and active caspase-3 were up-regulated whereas PCNA and cyclinD1 were down-regulated with the increase of irradiation time, when compared to the sh-NC group. The influences of HULC knockdown on alterations of these proteins in LNCaP cells were consistent with that in PC3 cells (Figure 2B). Colorimetric results presented that caspase-3 activity in PC3 and LNCaP cells at 48 h post-irradiation were dramatically elevated by HULC knockdown compared with the sh-NC group (P<0.05 or P<0.01, Figure 2C). After 48 h of irradiation, HULC knockdown induced a prominent G0/G1 arrest in PC3 cells (Figure 2D) whereas HULC overexpression arrested cell cycle at S phase in PC3 cells (Figure 2E). Thus, we concluded that alterations of cell apoptosis and proliferation, induced by irradiation, were both effectively enhanced by HULC knockdown but were decreased by HULC overexpression.

### Resistance to irradiation of DU-145 cells was enhanced by HULC overexpression

**Figure 3.**
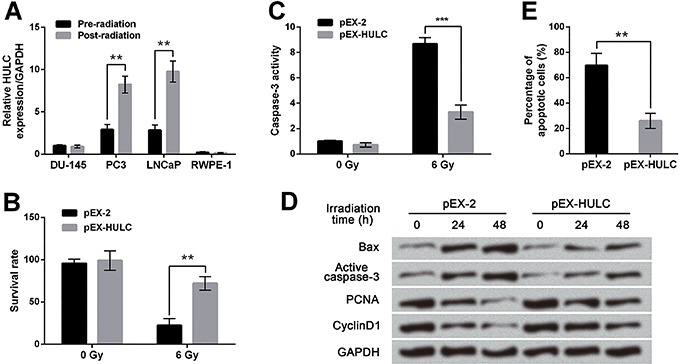
Resistance to irradiation of DU-145 cells was enhanced by HULC overexpression. *A*, DU-145 was sensitive to irradiation. *B*, Survival rate of DU-145 cells after irradiation was increased by HULC overexpression. *C*, Caspase-3 activity of DU-145 cells after irradiation was reduced by HULC overexpression. *D*, After irradiation, expressions of cell cycle-associated proteins were up-regulated while expressions of apoptosis-associated proteins were down-regulated by HULC overexpression. Data are reported as mean±SD. **P<0.01; ***P<0.001 (*t*-test). HULC: long non-coding RNA highly upregulated in liver cancer; pEX-HULC: pEX-2 plasmid carrying full-length HULC; PCNA: proliferating cell nuclear antigen.

After irradiation, expressions of HULC in PC3, LNCaP, DU145, and RWPE-1 cells were estimated. In Figure 3A, HULC expressions in PC3 and LNCaP cells were dramatically up-regulated after irradiation compared with that before radiation (both P<0.01), suggesting that DU-145 cells might be sensitive to irradiation. The survival rate of DU-145 cells overexpressing HULC after irradiation (6 Gy) was markedly higher than that of DU-145 cells transfected with pEX-2 (P<0.01, Figure 3B). Conversely, caspase-3 activity of HULC-overexpressed DU-145 cells was significantly lower than that of the pEX-2 group at 48 h post-irradiation (P<0.001, Figure 3C). Western blot analysis showed that irradiation-induced alterations of Bax, active caspase-3, PCNA, and cyclinD1 were all decreased by HULC overexpression at 24 and 48 h post-irradiation (Figure 3D). The percentage of apoptotic cells after 48 h of irradiation in HULC-overexpressed DU-145 cells was dramatically lower than that in pEX-2 transfected cells (P<0.01, Figure 3E). These results illustrated that resistance to irradiation of DU-145 cells was enhanced by HULC overexpression.

### Sensitivity of PC3 xenografts to irradiation was enhanced by HULC knockdown

**Figure 4.**
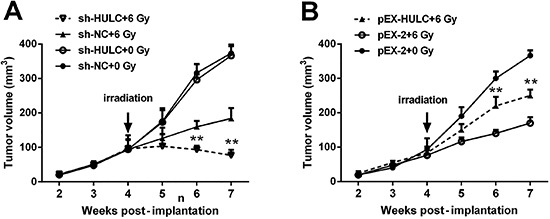
HULC knockdown enhanced the sensitivity of PC3 xenografts to irradiation. Tumor volume after irradiation was reduced by HULC knockdown (*A*) but was increased by HULC overexpression (*B*). Data are reported as mean±SD. **P<0.01 (*t*-test). HULC: long non-coding RNA highly upregulated in liver cancer; sh-HULC: pGPU6/GFP/Neo plasmid carrying short-hairpin RNA targeting HULC; sh-NC: pGPU6/GFP/Neo plasmid carrying a non-targeting RNA; pEX-HULC: pEX-2 plasmid carrying full-length HULC.

To explore the effects of HULC on tumor formation *in vivo*, NOD-SCID mice received xenografts of stably transfected PC3 cells. In groups that received 6 Gy irradiation, tumor volume in mice implanted HULC-silenced PC3 cells was significantly smaller than the sh-NC group at 6 d and 7 d post-implantation (both P<0.01, Figure 4A). Moreover, under irradiation (6 Gy), tumor volume in mice implanted HULC-overexpressed PC3 cells was markedly larger than the pEX-2 group at 6 d and 7 d post-implantation (both P<0.01, Figure 4B). Data illustrated that sensitivity of PC3 xenografts to irradiation was enhanced by HULC knockdown.

### HULC inhibited Beclin-1 phosphorylation and autophagy through regulating the mTOR pathway

**Figure 5.**
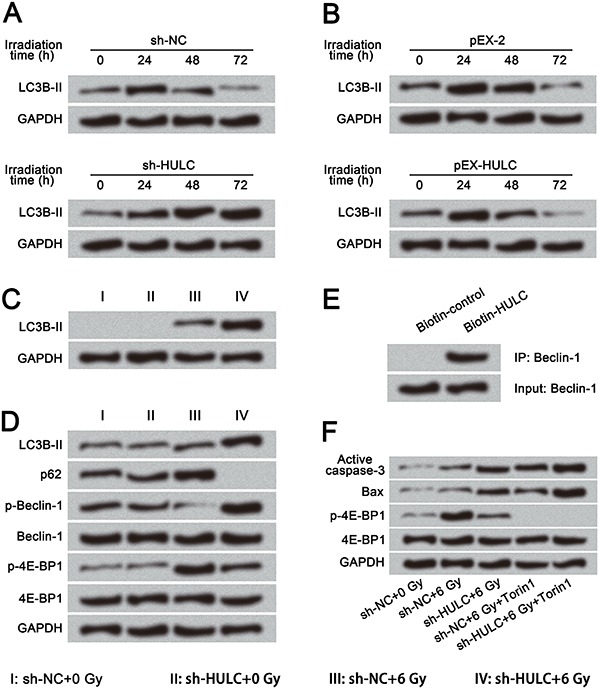
HULC inhibited Beclin-1 phosphorylation and autophagy through the mTOR pathway. Expression of LC3B-II after irradiation was up-regulated by HULC silence (*A*) while down-regulated by HULC overexpression (*B*). *C,* Expression of LC3B-II in tumor formed from HULC silenced-PC3 cells was higher than the sh-NC group after irradiation. *D,* HULC knockdown promoted autophagy through suppressing the mTOR cascade. *E*, HULC bound to Beclin-1. *F*, The effect of HULC knockdown on PC3 cells was further augmented by mTOR inhibitor. HULC: long non-coding RNA highly upregulated in liver cancer; sh-HULC: pGPU6/GFP/Neo plasmid carrying short-hairpin RNA targeting HULC; sh-NC: pGPU6/GFP/Neo plasmid carrying a non-targeting RNA; pEX-HULC: pEX-2 plasmid carrying full-length HULC; LC3B-II: microtubule-associated protein 1 light chain 3B II; 4E-BP1: eukaryotic initiation factor 4E binding protein 1; p: phosphorylated.

The expression of the autophagy marker LC3B in stably transfected PC3 cells after 0, 24, 48, and 72 h of irradiation was assessed. In the sh-NC and pEX-2 groups, which acted as controls groups, LC3B-II was up-regulated at 24 and 48 h post-irradiation and then down-regulated at 72 h post-irradiation (Figure 5A and B). However, the expression of LC3B-II was persistently up-regulated after irradiation in HULC-silenced PC3 cells, whereas LC3B-II expression in HULC-overexpressed cells was down-regulated at 48 and 72 h after irradiation; the LC3B-II expression was even lower than the control group. The LC3B-II expression in tumor from mice implanted sh-HULC or sh-NC transfected cells was also tested, and the results in Figure 5C showed LC3B-II expression was up-regulated by HULC knockdown after irradiation. Further studies also showed that LC3B-II and p-Beclin-1 were up-regulated whereas p62 and p-4E-BP1 were down-regulated by HULC knockdown at 72 h post-irradiation (Figure 5D). Subsequently, RNA-pull down assay illustrated that Beclin-1 was pulled down by Biotin-HULC using western blot analysis (Figure 5E). In Figure 5F, expressions of active caspase-3 and Bax were up-regulated while p-4E-BP1 expression was down-regulated by HULC knockdown after irradiation. However, with the presence of Torin 1, p-4E-BP1 was totally inhibited and the expressions of active caspase-3 and Bax were both up-regulated. Taken together, we concluded that HULC knockdown elevated phosphorylated levels of Beclin-1, and thereby promoted autophagy through inhibition of the mTOR pathway.

## Discussion

In our study, the roles as well as related mechanisms in the modulation of HULC for the resistance to irradiation in PCa cells were explored. Radiation-induced decreases of survival rate in PCa cells were further decreased by HULC knockdown while reversed by HULC overexpression. The cell cycle was repressed whereas cell apoptosis was promoted by HULC knockdown in PCa cells under irradiation. The sensitivity of DU-145 cells to irradiation was reduced by HULC overexpression. *In vivo* tumor formation experiments proved the modulation of HULC on sensitivity of PCa cells to irradiation. For the mechanism study, HULC could bind to Beclin-1 and then inhibited phosphorylation of Beclin-1, thereby inhibiting autophagy. Silenced HULC promoted autophagy along with inhibition of the mTOR cascade after irradiation, resulting in increased cell apoptosis. Moreover, the effects of HULC knockdown were further enhanced by the mTOR inhibition.

HULC has been reported to be a carcinogenic factor in multiple cancers. For example, Lu et al. (22) showed that HULC promoted cell proliferation in chronic myeloid leukemia. Zhao et al. (23) reported that HULC promoted cell proliferation and invasion but suppressed cell apoptosis in gastric cancer. However, the effects of HULC on PCa remain unclear. In the present study, the irradiation-induced decrease of survival rate in PCa cells and alteration of survival rate induced by irradiation were further augmented by HULC knockdown but reduced by HULC overexpression. Results illustrated that sensitivity to irradiation of PCa cells was enhanced by HULC knockdown.

Next, alterations of cell cycle and cell apoptosis as well as expression of associated proteins were assessed. Bax, a pro-apoptotic member of Bcl-2 family, is critical for cell survival and death (24). Bax promotes cell apoptosis by inducing the release of pro-apoptotic proteins, which initiate a caspase cascade (25). Active caspase-3 contributing to DNA fragmentation and protein degradation is the executioner of cell apoptosis (26). Thus, we tested the alteration of these two proteins as well as caspase-3 activity to estimate the change of cell apoptosis. Results showed that HULC knockdown promoted irradiation-induced cell apoptosis. PCNA is recognized as a marker for proliferation by adopting a ring-shaped structure to facilitate DNA synthesis during DNA replication (27). CyclinD1 is an important promoter of cell cycle, promoting progression from G1 to S-phase (28). In our study, results proposed that HULC knockdown is associated with enhanced sensitivity to irradiation of PCa cells by down-regulating PCNA and cyclinD1 and up-regulating Bax and active caspase-3.

As HULC silence could enhance sensitivity to irradiation, HULC levels in three PCa cell lines and a normal cell line were assessed. Results showed that HULC level in DU-145 cells was significantly lower than the other two PCa cell lines, indicating DU-145 might be most sensitive to irradiation. Then, DU-145 cells were transfected with pEX-2 or pEX-HULC, followed by assessments of caspase-3 activity, cell apoptosis, survival rate, and expressions of apoptosis- and cell cycle-associated proteins. Results illustrated that sensitivity to irradiation of DU-145 cells was reduced by HULC overexpression.

In addition to *in vitro* experiments, effects of HULC on irradiation-treated PC3 cells *in vivo* were also investigated. PC3 cells aberrantly expressing HULC and respective controls were implanted into mice. Tumor volume was reduced dramatically after irradiation and the group’s rank was pEX-HULC group<control groups<sh-NC group, suggesting that sensitivity to irradiation of PC3 cells *in vivo* was enhanced by HULC knockdown but was decreased by HULC overexpression, which was consistent with the *in vitro* study.

A previous study has proposed that enhanced autophagy improves the radiation sensitivity of PCa cells (29). Therefore, to explore the underlying mechanism, the alterations of autophagy were tested through LC3B-II expression as lipidated LC3B-II is critical for the formation of autophagosome (30,31). In our study, LC3B-II level was up-regulated by HULC silence and down-regulated by HULC overexpression, indicating that HULC inhibited autophagy after irradiation, which was verified *in vivo*. During autophagy, ubiquitinated proteins were targeted to autophagosome via p62; p62 degradation was another marker of autophagy (32,33). Phosphorylation of Thr119 of Beclin-1 causes dissociation of Bcl-2 from Beclin-1 resulting in autophagy activation (34). Autophagy is regulated by diverse pathways, including the mTOR cascade (35). In the present study, HULC interacted with Beclin-1 and its knockdown led to increase of Beclin-1 phosphorylation, along with inhibition of the mTOR pathway.

To summarize, HULC knockdown improved sensitivity of PCa cells to irradiation by promoting cell apoptosis and autophagy and inhibiting cell cycle both *in vivo* and *in vitro*. For the mechanism study, we found that HULC bound to Beclin-1 and thereby suppressed phosphorylation of Beclin-1, resulting in reduced autophagy through modulation of the mTOR pathway. This study provided an innovative strategy for the resistance to irradiation in the treatment of PCa, which is of great importance for its clinical treatment.
